# Impact of At-Home Telemonitoring on Health Services Expenditure and Hospital Admissions in Patients With Chronic Conditions: Before and After Control Intervention Analysis

**DOI:** 10.2196/medinform.7308

**Published:** 2017-09-08

**Authors:** Branko Celler, Marlien Varnfield, Surya Nepal, Ross Sparks, Jane Li, Rajiv Jayasena

**Affiliations:** ^1^ Biomedical Systems Research Laboratory University of New South Wales Sydney, NSW Australia; ^2^ Health and Biosecurity Business Unit eHealth Research Program Commonwealth Scientific and Industrial Research Organisation Herston QLD Australia; ^3^ Data 61, Software and Computational Systems Program Commonwealth Scientific and Industrial Research Organisation Marsfield, NSW Australia; ^4^ Health and Biosecurity Business Unit eHealth Research Program Commonwealth Scientific and Industrial Research Organisation North Ryde, NSW Australia; ^5^ Health and Biosecurity Business Unit eHealth Research Unit Commonwealth Scientific and Industrial Research Organisation Marsfield, NSW Australia; ^6^ Health and Biosecurity Business Unit eHealth Research Program Commonwealth Scientific and Industrial Research Organisation Parkville, VIC Australia

**Keywords:** telehealth, telemonitoring, chronic disease management, healthcare outcomes, BACI analysis

## Abstract

**Background:**

Telemonitoring is becoming increasingly important for the management of patients with chronic conditions, especially in countries with large distances such as Australia. However, despite large national investments in health information technology, little policy work has been undertaken in Australia in deploying telehealth in the home as a solution to the increasing demands and costs of managing chronic disease.

**Objective:**

The objective of this trial was to evaluate the impact of introducing at-home telemonitoring to patients living with chronic conditions on health care expenditure, number of admissions to hospital, and length of stay (LOS).

**Methods:**

A before and after control intervention analysis model was adopted whereby at each location patients were selected from a list of eligible patients living with a range of chronic conditions. Each test patient was case matched with at least one control patient. Test patients were supplied with a telehealth vital signs monitor and were remotely managed by a trained clinical care coordinator, while control patients continued to receive usual care. A total of 100 test patients and 137 control patients were analyzed. Primary health care benefits provided to Australian patients were investigated for the trial cohort. Time series data were analyzed using linear regression and analysis of covariance for a period of 3 years before the intervention and 1 year after.

**Results:**

There were no significant differences between test and control patients at baseline. Test patients were monitored for an average of 276 days with 75% of patients monitored for more than 6 months. Test patients 1 year after the start of their intervention showed a 46.3% reduction in rate of predicted medical expenditure, a 25.5% reduction in the rate of predicted pharmaceutical expenditure, a 53.2% reduction in the rate of predicted unscheduled admission to hospital, a 67.9% reduction in the predicted rate of LOS when admitted to hospital, and a reduction in mortality of between 41.3% and 44.5% relative to control patients. Control patients did not demonstrate any significant change in their predicted trajectory for any of the above variables.

**Conclusions:**

At-home telemonitoring of chronically ill patients showed a statistically robust positive impact increasing over time on health care expenditure, number of admissions to hospital, and LOS as well as a reduction in mortality.

**Trial Registration:**

Retrospectively registered with the Australian and New Zealand Clinical Trial Registry ACTRN12613000635763; https://www.anzctr.org.au/Trial/Registration/TrialReview.aspx?id=364030 (Archived by WebCite at http://www.webcitation.org/6sxqjkJHW)

## Introduction

In industrialized nations, approximately 70% to 78% of health care budgets are spent on the management of chronic disease or its exacerbation [1]. As the population ages the burden of chronic disease will increase and place health care budgets under increasing strain [2,3]. Telehealth services, with at-home telemonitoring of vital signs, have been demonstrated to deliver cost effective, timely, and improved access to quality care [4-7]. These services also reduce social dislocation and enhance the quality of life by allowing chronically ill and aged members to stay in their homes and communities longer [6-7].

One of the largest trials for evaluating telehealth outcomes was the Whole System Demonstrator (WSD) trial in the United Kingdom [8-10]. Results from this trial have been extensively reported and showed reductions in hospital admissions, bed days, costs, and mortality. However, experience in Australia with the deployment of at-home telemonitoring services is very limited [11-12]. Most trials are small scale and lack detailed analysis of key success factors such as health care outcomes, health economic benefits, impact on clinical work force availability, and acceptability by patients, carers, nurses, primary care physicians (PCP), and health care managers as well as the effect of workplace culture and capacity for organizational change management [13].

Despite large national investments in health information technology, very little policy work has been undertaken in Australia in deploying at-home telemonitoring as a solution to the increasing demands and costs of managing chronic disease.

This trial was designed to develop a robust evidence base for a number of key factors and demonstrate an effective and scalable model for Internet-enabled at-home telemonitoring services in Australia. Armed with the insights provided by this evidence base, policy makers may have much of the data they require to implement funding models and create a sustainable telehealth services sector in Australia.

## Methods

### Research Ethics Committee Approval

The clinical trial protocol for this study was approved by the Commonwealth Scientific and Industrial Research Organisation (CSIRO) Human Research Ethics Committee (HREC) (Approval Number 13/04, March 25, 2013) as well as 5 other local HRECs. A journal article on the clinical trial design has been previously published [13].

### Patient Selection and Recruitment

A before and after control intervention (BACI) design was used where control patients were matched to each test patient. This design [13-16] is well known in environmental intervention studies but is less known for health interventions. However, it has theoretical justifications for studies involving heterogeneous populations and has been successfully applied in many environmental intervention studies [14-16].

Candidates were eligible to participate in the study if they met inclusion criteria which were comprehensively described in an earlier publication [13] but are mentioned here briefly for convenience: age 50 years and older; 2 or more unplanned acute admissions during the last 12 months or 4 or more unplanned acute admissions during the previous 5 years, with a principal diagnosis of chronic obstructive pulmonary disease, coronary artery disease, hypertensive diseases, congestive heart failure, diabetes, or asthma. Eligible patients could be under the care of a community nurse or PCP or participants in a government care program other than special targeted programs to support individuals with high-care needs. Patients were also excluded if they were diagnosed with compromised cognitive function [17], a neuromuscular disease, or a psychiatric condition.

For each test participant, as many as 4 control candidates were automatically case matched [13] on gender, age, chronic condition, and socioeconomic indexes for areas (SEIFA) [18]. On their consent, the 2 closest matching control candidates commenced as participants in the study. We noted that in many cases only 1 acceptable match was available. When a test patient had more than 1 control, the data for the matched control patients were averaged to obtain a single matched pair. Both test patients and control patients continued to receive normal care under the management of their PCP.

**Figure 1:**
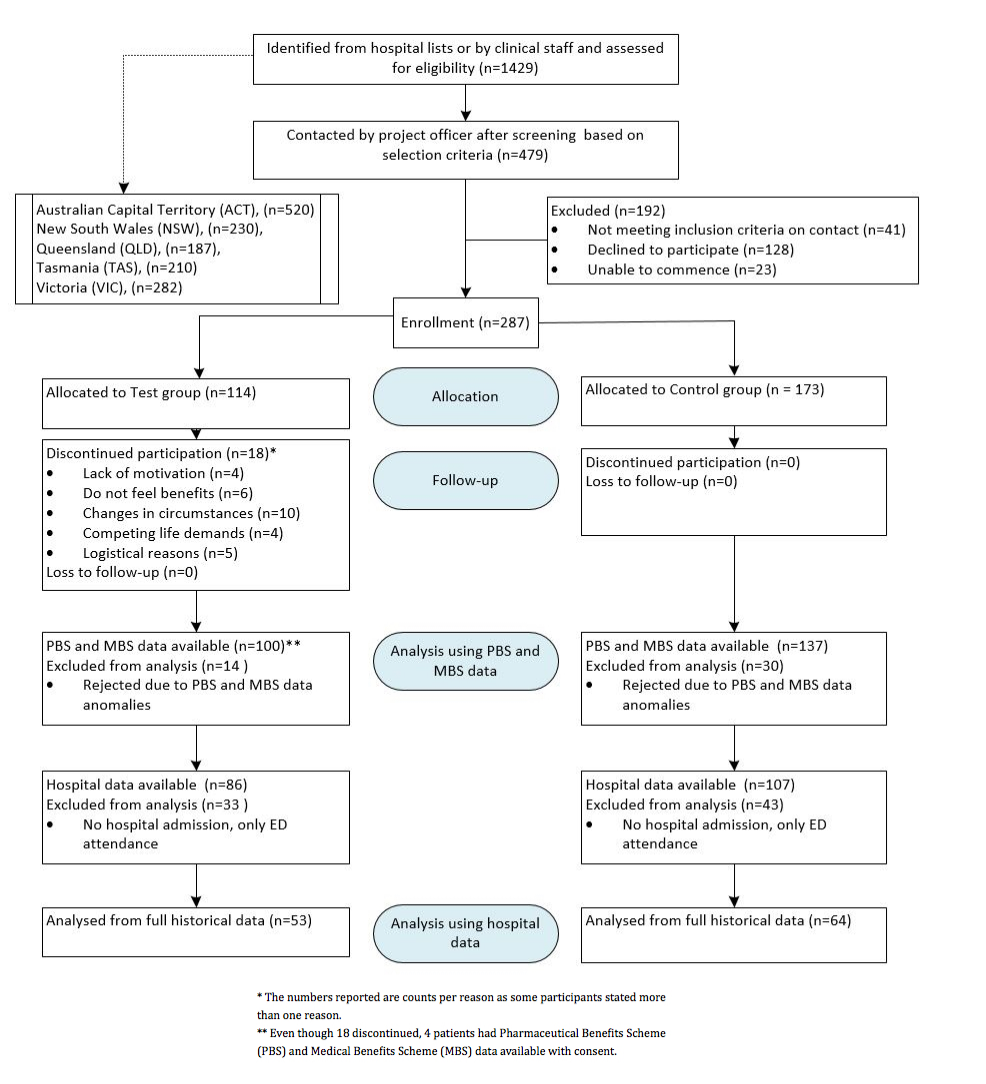
Recruitment flow chart.

Figure 1 shows the recruitment process and flow of participants through the study. A total of 1429 eligible patients were identified from hospital lists provided by local health districts and patients known to clinical staff. From these, 479 were still deemed eligible following individual screening and were contactable.

Following exclusions, a master list of 114 test patients and 173 control patients, all with pharmaceutical benefit scheme (PBS) and medical benefit scheme (MBS) data, was formed. On careful analysis of these data made available from the Australian Government Department of Human Services (DHS), it was observed that some patients had missing data. As a result, data from a number of test patients and control patients were rejected from further analysis. This led to a final matched cohort of 100 test patients and 137 control patients.

Hospital data were intended to be sourced for all test and control patients selected from hospital lists at each of the 5 test sites. However, as some test and control patients were not selected from hospital lists, their hospital data were thus not available for analysis. From the 100 test and 137 control patients matched for analysis of medical and pharmaceutical benefits data, 86 and 107, respectively, were matched for analysis of hospital admission and length of stay (LOS).

On detailed analysis of available patient hospital data, it was found that some patients had attended the emergency department of their local hospital, in some cases more than once on the same day, without being admitted. As a result, we decided to count an admission as involving at least 1 overnight stay, and this led to the further rejection of 33 test patients and 43 control patients, who based on these criteria, had no admissions to hospital.

This resulted in a final cohort of 53 test and 64 control patients for which full historical hospital data were available.

### Organization of Care

A project officer (PO) at each test site was responsible for managing operational and research activities for the study, thereby separating patient care from study operations. Test patients were supplied with the Telemedcare Telemonitoring Unit (TMU) by the PO who also trained them on its use [13]. The PO was also responsible for consenting patients, onsite visits, equipment maintenance, and technical support.

The clinical care coordinator (CCC) monitored patient vital signs and clinical questionnaire responses recorded via the Telemedcare TMU daily during business hours. The CCCs were experienced nursing staff, seconded part-time from each trial site health service provider. Their role was to coordinate the delivery of care when the telemonitoring of vital signs data and follow-up contact with patients indicated that they were experiencing an exacerbation of their condition. Normal care for the majority of test patients was by their local PCP.

Participants in the test group were provided with the Telemedcare TMU which was configured by the site PO or CCCs to reflect clinical best practice for the patient’s clinical condition. Patients would be reminded to record their vital signs measurements (such as blood pressure, oxygen saturation, electrocardiogram, spirometry, temperature, weight, and blood glucose), scheduled at a convenient time, typically in the morning before taking their medications.

Control participants received care as usual according to the service model of the respective trial site. They had no further contact with the PO after the consent process.

### Comparison of Medical and Pharmaceutical Expenditure Before the Start and Close to the End of the Trial

In order to compare the statistical match of test and control patients with respect to medical and pharmaceutical expenditure at the onset of telemonitoring, individual costs were summed over a period of 100 days just prior to the beginning of the intervention and in the last 100 days prior to the end of the intervention. The paired *t* test was then used to identify significant changes between test and control patients in both time periods.

### Regression Modeling

Medical, pharmaceutical, and hospital data were all synchronized to the date when the telemonitoring commenced to average out seasonal effects. Medical and pharmaceutical cost data for every patient were summed over 36 30-day periods before the start of the trial and 12 30-day periods after the start of the trial. This approximates analyzing data over 3 years before and 1 year after the start of telemonitoring.

Hospital admissions and LOS data were similarly treated, except that the time interval chosen was 100 days. This was a preferred interval as hospital admissions were much less frequent and would otherwise generate data with a large number of zero entries.

All the outcome variables were expected to increase over time because all patients were chronically ill and aging. We fitted a linear model including the explanatory variables 30-day or 100-day time period number, before-after indicator variable, and the interaction between these two variables.

To carry this out, the outcome variables of all test and control patients were averaged within each time period number. Normality of data was tested in each outcome and where necessary, square root (sqrt) or LogNormal transforms were applied.

Before and after data were analyzed, both as separate time period numbers with different slopes or as 1 time period having the same slope. This analysis was applied to (1) test patient data, (2) control patient data, and (3) difference (control-test) data.

These time series analyses permitted the determination of how well test patients and control patients were indeed matched, controlled for possible effects of the intervention on control patients by also analyzing differences (control-test) and reduced possible seasonal and other time varying influences.

Sqrt transformation was applied to medical and pharmaceutical benefits data before linear regression analysis was carried out. This was repeated both for test patient data and control patient data. Difference data calculated from control-test values for each data point were found to be normally distributed and did not need the application of any transform.

The time course of before and after data was modeled using linear regression and analysis of covariance (ANCOVA) analysis of slopes to identify statistically significant differences in before and after slopes, using the differences (control-test) to test and validate the results.

To estimate savings in expenditure over the year following the start of intervention, sqrt (30-day costs) were converted to annual costs by multiplying each 30-day data point by 365/30 and each 100 day data point by 365/100.

As a result of sqrt normalization, the functions for medical and pharmaceutical costs before and after intervention become quadratic, and estimates of savings require the calculation of predicted costs 1 year after the start of intervention based on the projection of the 3-year historical trajectory, 1 year past the start of intervention. The total predicted medical benefits expenditure for the year following intervention was estimated from the area under the annual expenditure curve projected 1 year from the start of intervention.

Following intervention, we would expect the slope of the regression line to change, and the area of the curve beneath the actual expenditure curve then provides an estimate of the actual expenditure for that year. The difference in the 2 areas is an estimate of savings over the year.

Linear regression was carried out using the fit command in the MATLAB (The MathWorks Inc) statistics toolbox. Outliers were excluded from the linear regression. The command predObs was used to plot 95% prediction intervals. Prediction intervals indicate a 95% probability that a future observation at x will fall within its boundaries.

Standard goodness of fit measures, including the sum of squares due to error, the coefficient of determination (R2), the R2 value adjusted for degree of freedom, and the standard error or root mean square error were also available. The control-test difference data were similarly analyzed.

### Estimating Mortality

A master register (MR) file of 1429 patients was formed by combining the hospital records from each local health district in each state and territory. Deaths of patients in this master file were subsequently cross-checked against the records of the Births, Deaths, and Marriages Register (BDMR) in each state and territory.

To more accurately compare mortality between test and control groups, the effect of the population’s age distribution must be taken into account. We thus use age-specific death rates (ASDRs), defined as the ratio of the number of deaths in a given age group to the population of that age group. For both methods, we compare actual mortality data against ASDRs calculated from the master register of eligible patients.

### Statistical Analysis

Comparisons, using the cases available, were made between the 2 groups at baseline using the chi-square test or Fisher exact test for categorical variables, the 2-sample *t* test for continuous variables, and the Wilcoxon rank-sum test for skewed variables. Baseline characteristics for both test and control patients are described using mean and standard deviations (SD) for continuous symmetrical variables and medians and 95% confidence intervals (CI) for skewed data.

Categorical variables are presented as counts and percentages. Within matched group differences (matched control minus test data) from baseline to last point were examined using the paired *t* test for symmetrical data and the Wilcoxon signed-rank test for skewed data. All statistical tests were 2-tailed, and a *P* value of <.05 was used to indicate statistical significance. Statistical analysis was performed using Stata verison 12 (StataCorp LLC), SPSS version 17 (IBM Corp), Matlab R2015b (The MathWorks Inc), and Excel (Microsoft Corp).

A data integration engine [18] facilitated the generation of various graphs using structured query language queries and either built-in Excel graph functions or Visual Basic programming for more complex graphs.

## Results

There were no significant differences in age between test (71.1 [SD 8.7] years; n=100) and control (71.7 [SD 9.0] years; n=137) patients or between male and female patients. There were also no statistical differences observed between test and control patients with respect to their SEIFA status or their primary disease diagnosis.

A total of 67% (67/100) of the test patients were male and 33% (33/100) were female. For control patients, 43.8% (60/137) were female and 56.2% (77/137) were male. Most patients had more than 1 condition listed as a primary diagnosis. For simplicity, primary disease conditions were grouped in the broad categories of cardiovascular disease (N_Test_=50), respiratory disease (N_Test_=30) and diabetes (N_Test_=20) although some patients had multiple comorbidities.

Test patients were monitored on average for 276 days, with no significant difference between average monitoring durations for female patients (266 days) and male patients (281 days). A total of 75% (75/100) of all test patients were monitored for periods exceeding 6 months.

Table 1 shows that there were no significant differences between test and control patients in terms of baseline total cost of medical and pharmaceutical benefits items for 100 days immediately preceding the start of intervention. However, for the last 100 days prior to the end of intervention, there was a significant difference in medical and pharmaceutical expenditure, with control patients spending on average $3298 more per year than test patients.

**Table 1:** Baseline comparison between test patients and control patients over 100 days prior to intervention and last 100 days prior to end of intervention.

Variable		Control patients $ (95% CI)	Test patients $ (95% CI)	*P* value
**Expenditure in last 100 days prior to start of intervention (N**_Test_**=100, N**_Control_**=137)**				
	Total cost of medications prescribed	975.8 (755-1205)	919.4 (748-1080)	.42
	Total expenditure on medical and pharmaceutical items	1931.7 (1525-2339)	2044 (1648-2423)	.12
**Expenditure in last 100 days prior to end of intervention (N**_Test_**=100, N**_Control_**=137)**				
	Total cost of medications prescribed	859.7 (615-1149)	505.9 (318-770)	<.001
	Total expenditure on medical and pharmaceutical items	1941.7 (1366-2637)	1038.2 (656-1570)	<.001

### Linear Regression Analysis

**Figure 2:**
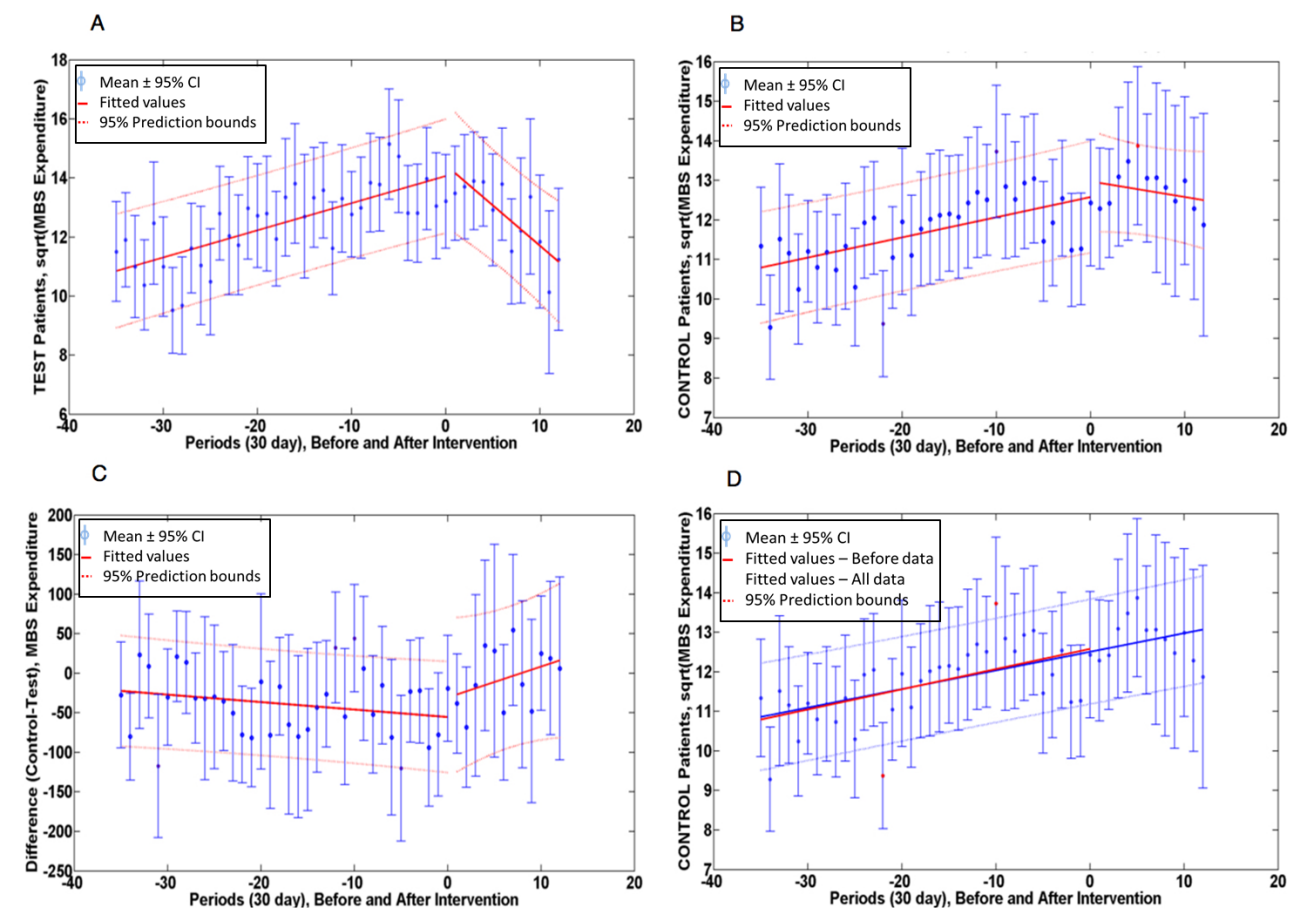
sqrt(MBS medical $ expenditure) plotted for (A) test patients and (B) control patients. Linear differences (control-test) are plotted in (C). Panel (D) shows change in regression line when before and after data are combined for control patients.

Figure 2 shows the medical costs averaged over 36 intervals of 30 days before and 12 intervals of 30 days after intervention. Figure 2 A and 2B show the sqrt($ medical expenditure) for test and control patients, Figure 2 C shows the linear difference in $ medical expenditure. Additional ANCOVA analysis comparing slopes for control patients of the combined before and after data as a single line to the before data alone, as shown in Figure 2 D, shows that there was no significant before and after difference (*P*=.929).

### Comparing Slopes Before Intervention

The plots shown above the linear regression fits and the results of the ANCOVA analysis for Figure 2 are given in tabular form in Multimedia Appendix 1, which also gives linear regression data for pharmaceutical expenditure, number of admissions to hospital, and hospital LOS.

Before the intervention, test patients and control patients had no significant difference in the rate of admission to hospital (*P*=.443), but test patients had a significantly greater rate of LOS (*P*=.013) in hospital.

### Comparing Before and After Slopes

For test patient medical expenditure, the slope before the intervention was significantly reduced (*P*<.001) following the intervention, indicating a significant reduction in the rate of medical expenditure. In contrast, for control patients the change in slope was not significant (*P*=.10).

For pharmaceutical expenditure, the fall in slope for test patients was highly significant (*P*<.001), while control patients showed a marginally significant (*P*=.046) increase in slope. As a result, the change in slope of the (control-test) difference was also significantly different (*P*=.008).

Control patients had no significant difference (*P*=.458) in their rate of admission to hospital before and after the intervention, while test patients had a significant fall (*P*=.009). Similarly, control patients had no significant change in LOS (*P*=.869), while test patients showed a significant (*P*=.006) fall in their LOS after the intervention, and the differences in (control-test) slopes before and after intervention were also significantly different (*P*<.001).

### Estimating Changes in the Rate of Expenditure and Savings After One Year

**Figure 3:**
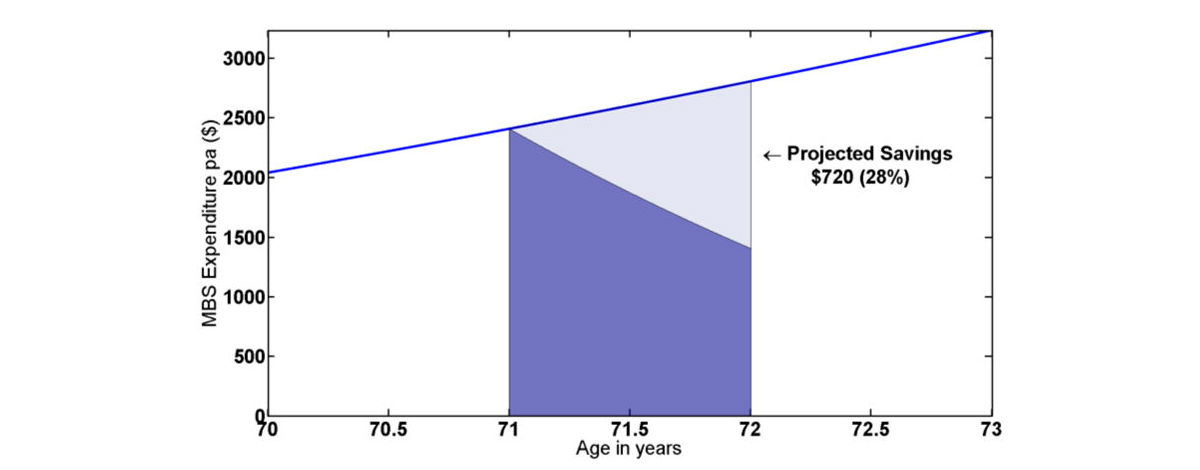
Model-based method of estimating impact of telemonitoring on medical expenditure.

Both the change in the rate of expenditure and savings in the year following the start of intervention for medical benefits or pharmaceutical benefits expenditure as well as number of admissions and LOS were estimated from the linear regression equations given in Multimedia Appendix 1. The method for estimating changes in rates and savings over the year is demonstrated in Figure 3 using medical benefits expenditure as an example.

The linear regressions for sqrt(30 day medical costs) developed for test patients, control patients, and differences (control-test) provide a best fit estimate of expenditure before and after intervention. The regression equations for data for 3 years prior to the intervention are projected forward by 1 year to estimate the predicted costs 1 year after the start of intervention.

This is shown in Figure 3 with some simplification for medical costs for all test patients. In Figure 3, the average age of test patients was approximately 71 years old at the start of intervention and was used as the reference point. The difference between the projected curve and the actual expenditure curve, representing the estimated saving over 1 year, was $720 or 28% of the projected expenditure.

**Figure 4:**
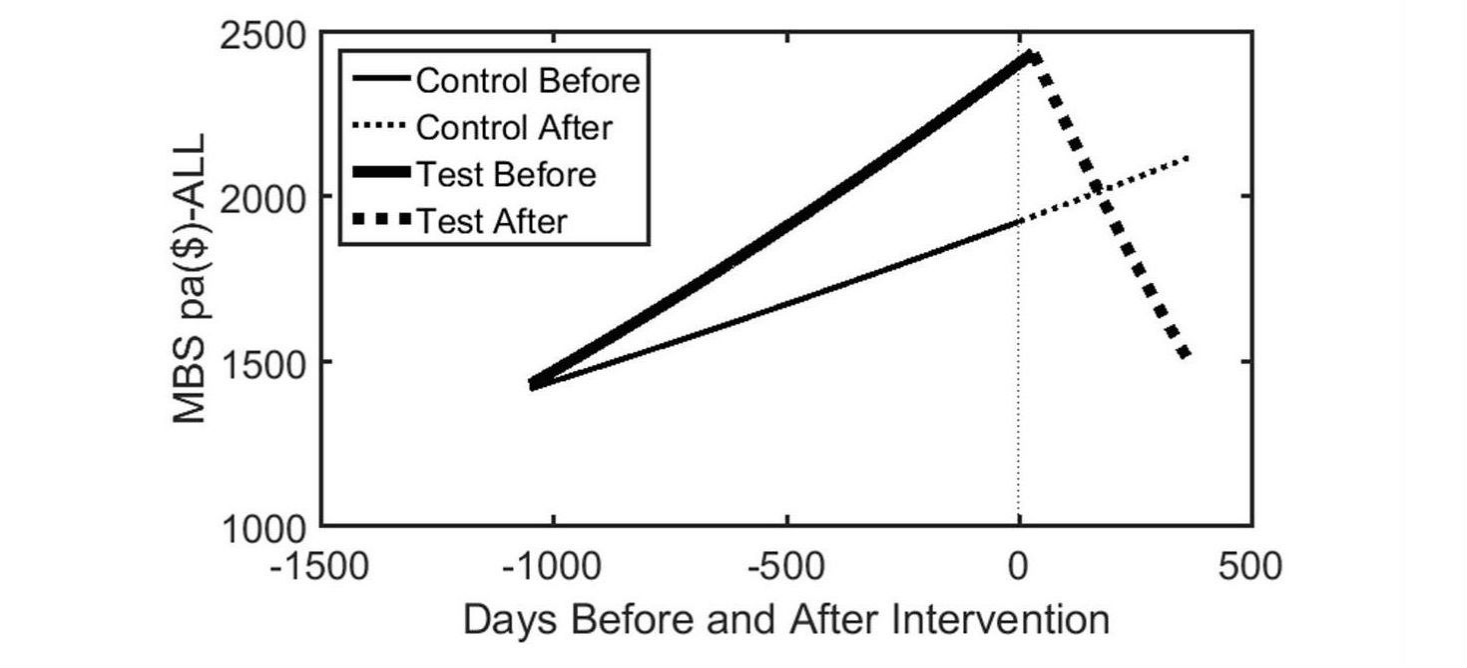
Regression-based estimates of time course of annual medical benefits expenditure for test patients and control patients, before and after intervention. Based on data presented in Multimedia Appendix 1.

However, the assumption that the 2 curves meet exactly at the onset of intervention is a simplification that may overestimate the savings. If indeed the impact of intervention needs some time to take effect, we would expect the point of intersection to fall sometime after the start of telemonitoring, subject to the variability of the expenditure data. This is in fact what was observed in the majority of cases as shown in Figure 4 for medical expenditure for all test patients.

Figure 4 shows that the curvilinear function for control patients before intervention was extended to the after period. For test patients, the intercept of the 2 curvilinear plots before and after intervention occurred at approximately 31 days after the start of intervention, leading to a reduced estimate of the savings in medical benefits expenditure from $720 to $611 per annum. Applying a similar analysis to the regression equation for differences (control-test) in Multimedia Appendix 1 (panel A) results in a similar estimate of savings of $657 per annum.

Estimates for the reduction in rates of expenditure for medical and pharmaceutical costs, number of admissions, LOS, and average savings over 1 year is given in Multimedia Appendix 2.

**Table 2:** Age-specific death rates of test patients.

	Age distribution					
	50-60	60-70	70-80	80-90	90-100	Total
Age distribution in MR^a^	180	310	441	414	84	1429
Age distribution, %	12.60	21.69	30.86	28.97	5.88	100.0
Deaths in MR^a^ , n	17	46	60	91	37	251
ASDR^b^ , %	9.44	14.84	13.61	21.98	44.05	17.56^c^
Test patients by age, n	41	31	14	13	1	100
Age specific deaths, n	1	2	4	1	0	8
Expected deaths, n	3.87	4.60	1.91	2.86	0.44	13.68
Deaths saved, n	2.87	2.60	–2.09	1.86	0.44	5.68

^a^MR: master register.

^b^ASDR: age-specific death rate.

^c^Crude death rate.

### Effect of Intervention on Mortality

A total of 57 test patients and 76 control patients in the study were from the MR of 1429 patients. The crude death rate was 8.8% (5/57) for test patients and 17.1% (13/76) for their matched controls, giving a reduction in mortality of 48.5%.

For the 100 test patients for whom survival data was accurately available through the BDMR in each state, the ASDR of the test patients relative to those from the MR file are given in Table 2.

Using ADSRs in Table 2 calculated from the MR of eligible patients, 13.68 deaths were expected but only 8.0 were recorded. This represents a saving of 5.68 lives, a reduction of 41.5%.

## Discussion

### Principal Findings

The results of this study demonstrate a statistically robust positive impact, increasing over time, of at-home telemonitoring on health care expenditure, number of admissions to the hospital, and LOS as well as a reduction in mortality.

Table 1 demonstrates that test patients and their controls were generally well matched with respect to expenditure on pharmaceutical and medical items at the start of intervention. However, in the last 100 days prior to the end of the intervention, test patients were spending on average $3298 less on medical and pharmaceutical items than control patients.

Multimedia Appendix 1 shows that for the 3 years before the intervention, there was a significant difference in slope between test patients and control patients for medical and pharmaceutical expenditure and hospital LOS but not for the number of hospital admissions.

Interestingly, the slope for medical expenditure prior to intervention was larger for test patients than control patients, but the slope for pharmaceutical expenditure was smaller for test patients than control patients, thus indicating that test patients were more likely than control patients to use medical services but were less likely to spend money on pharmaceutical prescription medications.

### Impact of Telemonitoring on Medical and Pharmaceutical Expenditure

The predicted rate of medical and pharmaceutical expenditure one year after the start of intervention was estimated as $2803 per annum and $3176 per annum, respectively (Multimedia Appendix 2). As a result of the telemonitoring intervention, these rates of expenditure fell to $1504 per annum and $2365 per annum, a reduction of 46.3% and 25.5%, respectively. Over the year of the intervention, average savings in medical and pharmaceutical costs were estimated as $611 and $354, or 23.5% and 11.5%, respectively. However, differences (control-test) data suggest that savings in pharmaceutical costs may be marginal.

### Impact of Telemonitoring on Hospital Admissions and Length of Stay

Test patients at the start of telemonitoring had a rate of LOS averaging 19.8 days, which after 1 year were projected to increase to 24.6 days per annum (Multimedia Appendix 2). Telemonitoring reduced the projected yearly rate of LOS after 1 year from 24.6 days per annum to 7.9 days per annum, a reduction of 67.9%. Over the year following the telemonitoring intervention, this leads to an average saving of 7.5 days or 33.8% in hospital stays relative to the 22.2 days predicted over that year without the intervention.

### Effect of Telemonitoring on Mortality

The crude death rate was 8.8% for test patients and 15.8% for their matched controls, giving a reduction in mortality of 48.5% (Table 2). A more accurate method based on comparison of ASDRs of 100 test patients (8 deaths) for whom survival data was accurately available against the expected ASDRs generated from a master registry of 1429 patients (13.64 deaths) indicated a reduction in mortality of 41.5%.

### Generalization of Trial Results

The project design was a multistate, multisite trial along the eastern seaboard of Australia, and because health service provision across the country in urban settings is relatively uniform because of Medicare, the government-funded universal health care system, we believe that results can be generalized to the broader urban Australian population but not necessarily to rural and remote locations or other countries with different health systems.

These results are broadly in agreement but more favorable than those reported for the UK WSD trial [8-10] or the US Veterans Health Administration (VHA) Care Coordination/Home Telehealth (CCHT) program [19].

The headline findings for the WSD [8-10] included a 15% reduction in accident and emergency visits, a 20% reduction in emergency admissions, a 14% reduction in elective admissions, a 14% reduction in bed days, an 8% reduction in tariff costs, and a 45% reduction in mortality rates.

The differences in the results reported in this study can be attributed to different protocols for patient selection (general practitioner selection vs selection of matched test and controls patients from hospital lists) as well as differences in the quality and mode of analysis of the available data.

Between July 2003 and December 2007, the VHA implemented a national home telehealth program, CCHT, that supports the care for veterans with chronic conditions in their homes as they age.

The technology adopted in VHA service was considerably different to the telemonitoring technology used in this study and included videophones, messaging devices, biometric devices, digital cameras, and telemonitoring devices. More importantly, the age distribution was considerably different and included participants as young as 20 and older than 80 years with a wider range of conditions including posttraumatic stress disorder, depression, and other mental health condition.

Routine outcomes analysis for performance measurement of health care resource utilization by CCHT patients involved comparing hospital admission data for patients during the year prior to enrollment into CCHT with the data from 6 months postenrollment. This cohort of patients had a 19.74% reduction in hospital admissions and 25.31% reduction in bed days of care (BDOC) following enrollment into the CCHT program. During the same time period, there was a decline of 4.6% in BDOC for all patients enrolled within VHA, which needs to be taken into account when interpreting this change. Given the size, complexity, and resourcing of this program and the comprehensive and systematic approach to the clinical, educational, technology, and business processes that constitute VHA’s CCHT model of care, it is impossible to make a formal comparison of results. However given the small size, tight control on eligibility, and the greater homogeneity of the study cohort in our study, it is not surprising that we report considerably better results.

### Limitations

Like all complex clinical trials this project suffered numerous setbacks. Some of the major issues that impacted execution of the trial and subsequent data analysis are as follows:

- A significant number of eligible patients, including 93 test and 33 control patients, declined to participate, while 27 of those who agreed to participate were not able to commence and 18 test patients withdrew after they had begun monitoring.
- We recruited and consented 114 test patients and 173 control patients, but of these, only 71 test patients and 110 control patients were from the hospital lists provided. This caused considerable difficulty in the reliable assessment of mortality and the analysis of hospital admissions and LOS.
- Of the 114 test patients consented, 14 had missing data in their DHS records and had to be removed from further analysis. Similarly, of the 173 test patients consented, only 137 patients had complete DHS data. No explanation was available from DHS as to why some patients had missing data in their records.
- Test patients were recruited and initiated telemonitoring over a long period of time so that while the average number of days that patients were monitored was 276 days, there was a considerable spread, from <100 days to >500 days. The period for analysis of the effect of telemonitoring was thus limited to 12 months as patient numbers rapidly fall and the data spread increases for periods >12 months.
- For some patients consented early in the trial, signed consent was provided only through June 2014. When the trial duration was extended to the end of December 2014, new consent forms for the extended period were not signed and as a result, DHS data for these patients were only available through June 2014.
- CCCs were typically registered nurses employed by the service providers participating in the study. Most did not have any previous experience with telemonitoring but all graduated from a 2-day intensive training program on how to use the telemonitoring equipment and how to interpret the clinical data recorded. On average, CCCs spent a little over 33 minutes per week reviewing individual patient data, suggesting that as their average patient load was 20 patients, they were employed in this role <30% full-time over the week. This is less than optimal for this critical role.
- Although test and control patients were generally well matched by primary diagnosis, number of hospital admissions, and SEIFA index across sites, it was later observed that historical rates of medical and pharmaceutical expenditure were not well matched at the start of telemonitoring, as shown in Figure 4. Since the historical rate of growth of medical expenditure may be a good proxy for the present level of severity of a patient’s chronic condition, future studies should consider using this variable to match test and control patients in addition to the matching criteria used in this study.

### Conclusion

At-home telemonitoring leads to a significant time-dependent reduction in expenditure on medical services, a reduction in the number of hospital admissions, and a reduction in LOS averaging 7.5 days per annum. Mortality of test patients relative to control patients was also reduced by between 41.5% and 48.5% over the period of the trial.

It is not possible from this study to separate the effect of care coordination and coaching by the CCC from the direct and exclusive impact of at-home telemonitoring and patient self-management. However, the data presented shows clearly that the impact of at-home telemonitoring increases almost linearly over the first year following the intervention.

One would envisage that the impact would inevitably plateau and possibly begin to rise with increasing age and morbidity; however, longer term studies are required to elucidate the impact of telemonitoring over longer time frames.

Further research is also required to understand why hospital admissions that were recorded could not be avoided. Did the available vital signs not provide a sufficient warning of an exacerbation or were these warning signs ignored or not acted upon in a timely fashion by the nurse coordinator or patient’s PCP?

A detailed cost-benefit analysis of at-home telemonitoring as well as organizational change management requirements and workplace cultural issues that need to be considered in delivering the services reported in this study will be reported separately.

However, a preliminary cost-benefit analysis based on an estimate of the cost of delivering the telemonitoring service—approximately Aud $2760 per annum (Aud $7.40 per day)—against potential savings of more than Aud $19,000 per annum based on average cost of one bed day of Aud $2051 provides a return of investment of approximately 6 times.

## Appendix

Multimedia Appendix 1 Linear regression and analysis of covariance for (1) sqrt(medical expenditure), (2) sqrt(pharmaceutical expenditure), (3) number of hospital admissions, and (4) hospital length of stay.

Multimedia Appendix 2 Estimated changes in medical and pharmaceutical expenditure, hospital admissions, and length of stay for test patients with and without intervention.

## Abbreviations

ANCOVA: analysis of covariance
ASDR: age-specific death rate
BACI: before and after control intervention
BDMR: Births, Deaths, and Marriages Register
BDOC: bed days of care
CCC: clinical care coordinator
CCHT: Care Coordination/Home Telehealth
CSIRO: Commonwealth Scientific and Industrial Research Organisation
DHS: Department of Human Services
HREC: Human Research Ethics Committee
LOS: length of stay
MBS: medical benefits scheme (medical expenditure)
MR: master register of eligible patients
PBS: pharmaceutical benefits scheme (pharmaceutical expenditure)
PCP: primary care physician
PO: project officer
R2: coefficient of determination
SEIFA: socioeconomic indexes for areas
sqrt: square root
TMU: telemonitoring unit
VHA: Veterans Health Administration

## References

[1] National Center for Chronic Disease Prevention and Health Promotion (2009). The Power of Prevention.

[2] Anderson G, Horvath J (2004). The growing burden of chronic disease in America. Public Health Rep.

[3] Australian Institute of Health and Welfare Health expenditure Australia 2011–12.

[4] Totten A, Womack D, Eden K, McDonagh M, Griffin J, Grusing S, Hersh W Telehealth: mapping the evidence for patient outcomes from systematic reviews.

[5] McBain H, Shipley M, Newman S (2015). The impact of self-monitoring in chronic illness on healthcare utilisation: a systematic review of reviews. BMC Health Serv Res.

[6] Brettle AJ, Brown TM, Hardiker NR, Radcliffe JN, Smith CL Telehealth: the effects on clinical outcomes, cost effectiveness and the patient experience: a systematic overview of the literature.

[7] Paré G, Jaana M, Sicotte C (2007). Systematic review of home telemonitoring for chronic diseases: the evidence base. J Am Med Inform Assn.

[8] Bower P, Cartwright M, Hirani S (2011). A comprehensive evaluation of the impact of telemonitoring in patients with long-term conditions and social care needs: protocol for the whole systems demonstrator cluster randomised trial. BMC Health Serv Res.

[9] Steventon A, Bardsley M, Billings J, Dixon J, Doll H, Beynon M, Hirani S, Cartwright M, Rixon L, Knapp M, Henderson C (2013). Effect of telecare on use of health and social care services: findings from the Whole Systems Demonstrator cluster randomised trial. Age Ageing.

[10] Steventon A, Ariti C, Fisher E, Bardsley M (2016). Effect of telehealth on hospital utilisation and mortality in routine clinical practice: a matched control cohort study in an early adopter site. BMJ Open.

[11] Bradford N, Caffery L, Smith A (2016). Telehealth services in rural and remote Australia: a systematic review of models of care and factors influencing success and sustainability.

[12] Medical Technology Association of Australia A telehealth strategy for Australia: supporting patients in the community.

[13] Celler BG, Sparks R, Nepal S, Alem L, Varnfield M, Li J, Jang-Jaccard J, McBride S, Jayasena R (2014). Design of a multi-site multi-state clinical trial of home monitoring of chronic disease in the community in Australia. BMC Pub Health.

[14] Petticrew M, Cummins S, Sparks L, Findlay A (2007). Validating health impact assessment: prediction is difficult (especially about the future). Environ Impact Asses.

[15] Morrison-Saunders A, Bailey J (2003). Practitioner perspectives on the role of science in environmental impact assessment. Environ Manage.

[16] Underwood A (1991). Beyond BACI: experimental designs for detecting human environmental impacts on temporal variations in natural populations. Mar Freshwater Res.

[17] Jitapunkul S, Pillay I, Ebrahim S (1991). The abbreviated mental test: its use and validity. Age Ageing.

[18] (2006). Information paper: an introduction to socio-economic indexes for areas (SEIFA).

[19] Darkins A, Ryan P, Kobb R, Foster L, Edmonson E, Wakefield B, Lancaster AE (2008). Care Coordination/Home Telehealth: the systematic implementation of health informatics, home telehealth, and disease management to support the care of veteran patients with chronic conditions. Telemed J E Health.

