# Supplementary material for: Impact of At-Home Telemonitoring on Health Services Expenditure and Hospital Admissions in Patients With Chronic Conditions: Before and After Control Intervention Analysis
Source: JMIR Med Inform. 2017 Sep 8;5(3):e29. doi: 10.2196/medinform.7308 (PMC5610354; doi:10.2196/medinform.7308)
Supplement: Multimedia Appendix 1 [file medinform_v5i3e29_app1.pdf]

Multimedia Appendix 1. Linear regression and ANCOVA analysis for (1) sqrt(medical expenditure), (2) sqrt(pharmaceutical expenditure), (3) number of hospital admissions, and (4) hospital LOS. Difference values are (control-test). Bracketed terms are 95% CI.

	BEFORE SLOPE	AFTER SLOPE	*P* value	BEFORE INTERCEPT	AFTER INTERCEPT
**A. Medical expenditure** (N_{Test} = 100, $N_{Control}$ = 137)<br>Data points averaged over 30 day intervals					
**√CONTROL**	0.0510$^{\alpha\gamma}$<br>(0.0293, 0.0727)	-0.0395$^{\alpha\eta}$<br>(-0.1305, 0.0515)	0.1$^{\alpha}$	12.58<br>(12.13, 13.02)	12.98<br>(12.29, 13.66)
**√TEST**	0.0919$^{\delta\gamma}$<br>(0.0625, 0.1213)	-0.2729$^{\delta\eta}$<br>(-0.4236, -0.1222)	<0.001$^{\delta}$	14.06<br>(13.47, 14.66)	14.44<br>(13.33, 15.55)
**P Value**	0.0268$^{\gamma}$	0.009$^{\eta}$			
**DIFF**	-0.9446$^{\varepsilon}$<br>(-2.073, 0.1839)	3.916$^{\varepsilon}$<br>(-3.251, 11.08)	0.1025$^{\varepsilon}$	-55.38<br>(-78.71, -32.05)	-30.91<br>(-83.66, 21.84)
**B. Pharmaceutical expenditure** (N_{Test} = 100, $N_{Control}$ = 143).<br>Data points averaged over 30 day intervals					
**√CONTROL**	0.0824$^{\alpha\gamma}$<br>(0.0671, 0.0976)	0.1584$^{\alpha\eta}$<br>(0.1012, 0.2155)	0.0462$^{\alpha}$	16.5<br>(16.19, 16.81)	15.39<br>(14.97, 15.81)
**√TEST**	0.0408$^{\delta\gamma}$<br>(0.0260, 0.0557)	-0.1717$^{\delta\eta}$<br>(-0.2361, -0.1074)	<0.001$^{\delta}$	15.66<br>(15.36 - 15.96)	16.03<br>(15.55, 16.5)
**P**	P < 0.001$^{\gamma}$	P < 0.001$^{\eta}$			
**DIFF**	3.392$^{\varepsilon}$<br>(2.337, 4.448)	11.06$^{\varepsilon}$<br>(4.842, 17.27)	<0.0084$^{\varepsilon}$	73.67<br>(52.11, 95.22)	6.51<br>(-39.23, 52.25)
**C. Number of admissions to hospital** (N_{Test} = 53, $N_{Control}$ = 64).<br>Data points averaged over 100 day intervals					
**CONTROL**	0.0311<br>(0.0137, 0.0485)	0.0661<br>(-0.0432, 0.1754)	0.4576	0.5463<br>(0.423, 0.6696)	0.2624<br>(-0.0368, 0.5617)
**TEST**	0.0402<br>(0.0215, 0.0588)	-0.1109<br>(-0.2592, 0.0374)	0.0094	0.6998<br>(0.5678, 0.8318)	0.8011<br>(0.3949, 1.207)
**P**	0.4429	0.0145			
**DIFF**	-0.0107<br>(-0.0295, 0.0082)	0.177<br>(0.1146, 0.2393)	0.0018	-0.1361<br>(-0.2727, 0.0005)	-0.5387<br>(-0.7095, -0.3678)
**D. Hospital LOS** (N_{Test} = 53, $N_{Control}$ = 64)<br>Data points averaged over 100 day intervals					
**CONTROL**	0.1452$^{\alpha\gamma}$<br>(0.0669, 0.2235)	0.1$^{\alpha\eta}$<br>(-1.836, 2.036)	0.869$^{\alpha}$	2.739<br>(2.186, 3.293)	2.785<br>(-2.516, 8.086)
**TEST**	0.3597$^{\delta\gamma}$<br>(0.2049, 0.5145)	-1.038$^{\delta\eta}$<br>(-2.791, 0.7141)	0.006$^{\delta}$	5.424<br>(4.329, 6.518)	5.957<br>(1.158, 10.76)
**P**	0.0125$^{\gamma}$	0.1339$^{\eta}$			
**DIFF**	-0.2145$^{\varepsilon}$<br>(- 0.3883, -0.0407)	1.138$^{\varepsilon}$<br>(0.5418, 1.735)	<0.01$^{\varepsilon}$	-2.685<br>(-3.914,-1.455)	-3.172<br>(-4.806, -1.539)
