# Supplementary material for: Impact of At-Home Telemonitoring on Health Services Expenditure and Hospital Admissions in Patients With Chronic Conditions: Before and After Control Intervention Analysis
Source: JMIR Med Inform. 2017 Sep 8;5(3):e29. doi: 10.2196/medinform.7308 (PMC5610354; doi:10.2196/medinform.7308)
Supplement: Multimedia Appendix 2 [file medinform_v5i3e29_app2.pdf]

Multimedia Appendix 2. Estimated changes in medical and pharmaceutical expenditure, hospital admissions, and LOS for test patients with and without intervention. Expenditure is in Australian dollars.

	Medical Expenditure	Pharmaceutical Expenditure	Number of Admissions	LOS (days)
Estimated rate per annum at start of intervention	$2,405	$2,984	2.55	19.8
Predicted rate per annum after one year (without intervention)	$2,803	$3,176	3.09	24.6
Estimated rate pa after one year (with intervention)	$1,504	$2,365	1.45	7.9
% Reduction in rate pa after one year (with intervention)	46.3%	25.5%	53.2%	67.9%
Predicted average over one year without intervention	$2,602	$3,080	2.82	22.2
Estimated average over first year of intervention	$1,991	$2,726	2.15	14.7
Average savings/reductions over first year of intervention	$611	$354	0.67	7.5
Average (%) savings over first year of intervention	23.5%	11.5%	23.8%	33.8%
